# An agent-based model of the Notch signaling pathway elucidates three levels of complexity in the determination of developmental patterning

**DOI:** 10.1186/s12918-018-0672-9

**Published:** 2019-01-14

**Authors:** Elaine R. Reynolds, Ryan Himmelwright, Christopher Sanginiti, Jeffrey O. Pfaffmann

**Affiliations:** 1 Biology Department, Lafayette College, Easton, PA 18042 USA; 2 Neuroscience Program, Lafayette College, Easton, PA 18042 USA; 3 Present address: 530 Foster Street, Apt #512, Durham, NC 27701 USA; 4 Present address: 780 Richie Hwy, Suite S-30, Severna Park, MD 21146 USA; 5 Computer Science Department, Lafayette College, Easton, PA 18042 USA

**Keywords:** Agent-based modeling, Notch signaling pathway, Self-patterning

## Abstract

**Background:**

The Notch signaling pathway is involved in cell fate decision and developmental patterning in diverse organisms. A receptor molecule, Notch (N), and a ligand molecule (in this case Delta or Dl) are the central molecules in this pathway. In early Drosophila embryos, these molecules determine neural vs. skin fates in a reproducible rosette pattern.

**Results:**

We have created an agent-based model (ABM) that simulates the molecular components for this signaling pathway as agents acting within a spatial representation of a cell. The model captures the changing levels of these components, their transition from one state to another, and their movement from the nucleus to the cell membrane and back to the nucleus again. The model introduces stochastic variation into the system using a random generator within the Netlogo programming environment. The model uses these representations to understand the biological systems at three levels: individual cell fate, the interactions between cells, and the formation of pattern across the system. Using a set of assessment tools, we show that the current model accurately reproduces the rosette pattern of neurons and skin cells in the system over a wide set of parameters. Oscillations in the level of the N agent eventually stabilize cell fate into this pattern. We found that the dynamic timing and the availability of the N and Dl agents in neighboring cells are central to the formation of a correct and stable pattern. A feedback loop to the production of both components is necessary for a correct and stable pattern.

**Conclusions:**

The signaling pathways within and between cells in our model interact in real time to create a spatially correct field of neurons and skin cells. This model predicts that cells with high N and low Dl drive the formation of the pattern. This model also be used to elucidate general rules of biological self-patterning and decision-making.

**Electronic supplementary material:**

The online version of this article (10.1186/s12918-018-0672-9) contains supplementary material, which is available to authorized users.

## Background

Decision making during development is a widely studied problem. Generally speaking, biologists have genetically dissected fate pathways to identify molecules that interact through extracellular receptors with other cells and their environment to alter their transcription patterns through signaling pathways. However, the route to cell fate is not straightforward and a detailed understanding of how groups of cells develop spatial relationships necessitates a systems-level approach.

One of the canonical pathways for fate decisions and patterning is the Notch pathway. This pathway is used in multiple tissue types and across evolutionary time; it is the most common pathway used by adjacent cells to make binary fate decisions [1]. The Drosophila early embryo is the best studied Notch pathway, where Notch (N), an extracellular receptor, and its ligand Delta (Dl) act to determine the fate of a sheet of ectodermal cells, with cells adopting either a neuronal or epidermal fate in a reproducible rosette spatial pattern [2, 3]. Cells with high levels of N protein assume an epidermal fate, while a low level of N produces the neuronal fate. This signaling pathway yields a reproducible geometry and consistent number of neural and epidermal cells, however it is unclear how the identical cellular pathways within each cell interact to create pattern within a group of cells.

**Fig. 1:**
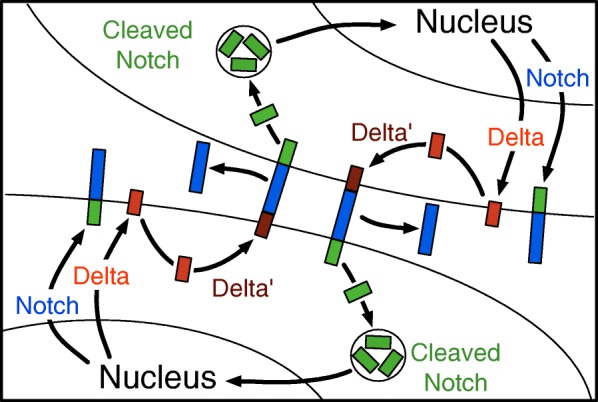
The cellular events of N signaling pathway. The steps are (1) N and Dl proteins are transcribed, translated and transported to the cell membrane; (2) Dl undergoes an endocytic processing step to become the ligand for N; (3) the Dl ligand and N receptor interact resulting in a cleavage and endocytosis of a N fragment; (4) A second cleavage releases a N product that is translocated to the nucleus

The N receptor, various ligands, modifying proteins, endocytic proteins, and transcriptional regulators have been identified and a detailed outline of the pathway has been established and is detailed in Fig. 1 [4, 5]. The signaling pathway begins with the transcription and translation of N and Dl proteins, which transit to the cell membrane (step 1). Dl undergoes an additional endocytic processing step and reappears on the surface as the ligand for N (step 2) [6, 7]. A heterodimeric interaction on adjacent cell surfaces occurs between the Dl ligand and N receptor resulting in a cleavage event and endocytosis of a N fragment (step 3). A second cleavage by presenilin releases a N product that is translocated to the nucleus (step 4) [8, 9]. This N fragment orchestrates, with several other transcription factors, the downstream regulation of genes associated with the epidermal fate. The no or low N alternative state also has transcriptional consequences that result in a neuronal fate. These addition factors also are responsible for stabilizing the fate of the cell [1, 4, 5]. There is only limited evidence that N regulates its own transcription or Dl transcription [10, 11]. Such feedback is thought by most investigators to be important to the population dynamics of the biological system and is characteristic of most regulatory circuits [1, 12, 13]. The Dl ligand doesn’t appear to have any downstream effects in the adjacent cell as the result of N binding, but its inactivation by cleavage after binding is important in the process [14]. While this system is often termed “lateral inhibition” the mechanism of the inhibition is not clear since Dl activates N, but does not have any inhibitory effect on the cell it is expressed in.

In addition to the molecular genetic work, mathematical and computational models of the N signaling have been created [15–25]. Many of these models uses equations or algorithms to represent the interactions between cells, although other approaches, such viewing the system as a dynamic network, have been utilized. These models replicate the in vivo pattern accurately and make important contributions to the understanding of the system, but model the development of fate choice within a single cell, or the dynamics between cells that results in the pattern within the system. No existing model captures the dynamics that connect the underlying molecular signaling pathway that produces the intercellular interactions to the overall patterning of cells during development.

We have built a fine-grained and hierarchical model using Agent-Based Modeling (ABM) that reproduces and assesses three levels of complexity: intracellular dynamics, intercellular interactions, and system dynamics [26–28]. Informed by the myriad molecular studies done with the Notch pathway, we created agents that mimic the actual cellular and protein components for a population of cells in a sheet and that undergo transformations with each cycle of the model. We believe it is necessary to include the intracellular components of the system, since the dynamics of these components set up the interaction between cells. The signaling pathways within and between cells in our model interact in real time to create a spatially correct field of neurons and skin cells. As agents interact with each other, an evolving dynamic picture of an emergent system can be observed and analyzed. We developed tools to track the stabilization of individual cell fate and the stable formation of pattern in the system and used these tools to evaluate the contributions of individual signal components to cell fate and system pattern. We altered both the levels and timing of these components using model features that control the production and transitions between agents. Our model suggests that cell fate and larger system pattern are generated through oscillations of the number of N agents and that out of sync oscillations created by the stochastic nature of the model produced alternative fates in neighboring cells. Each individual cell charts its own path to a cell fate choice, but interactions between cells cement those cell fates and the larger system pattern as the model progresses. The model replicates a number of features of the biological system and produces a number of testable hypotheses that can be explored further in both in silico and in vivo.

## Results

### Model development

To address the problem of multicellular patterning, we chose to model the N signaling pathway described above used since the current understanding of this pathway has broad experimental support [1]. An attractive aspect of using this signaling pathway in our modeling of this problem is that there is a single central signaling component, N, and its receptor. Both N and Dl are absolutely required for cell fate, cell interaction and the larger pattern. There is no cascade and the N signal in a cell is not amplified at any step, therefore the exact level of N protein controls cell fate. Using ABM, we can focus on one product, and add or control effectors in a stepwise fashion to see which are important to the overall patterning. We describe the basic features of the model below, with more details in the Methods.

Our agent-based model of the N signaling pathway constructed in a NetLogo modeling environment allows us to manipulate N and Dl and their transformations by representing them as model agents within a defined environment or space [29]. Typical of ABM techniques, there is a discrete abstraction of time where a single tick moves the model forward in its global progression. The model has two distinct families of agents, structural and active, that have different roles in the model. Agent names are **bolded** throughout the paper and defined in Table 1.

**Table 1:** Agents, their movements and transformations

Agent ID	Agent representation	Movement and/or transformation	Variable names within the code	Variable descriptions
**Nuc**	Cell nucleus	static	*nuc_radius*	Radius size around nucleus
**Mem**	Cell membrane	static	*radius lipid-density*	Cell radius and position # of lipid agents
**Dl**	Dl as initially transcribed	**Dl** moves towards **Mem** and converts to **Dlm**	*delta-transcription-initial-rate*	Chance that a single **Dl** will be produced
**Dlm**	Dl associated with membrane	Dlm moves laterally from **Mem** to **Mem** **Dlm** converts **Dlm’**	*delta-transform-time*	Time period before **Dlm** is converted to **Dlm’**
**Dlm’**	Dl form that interacts with N from another cell	moves laterally from **Mem** to **Mem** Ages out	*deltaAge*	Time period a **Dl** will exist
**N**	N as initially transcribed	**N** moves towards **Mem** and converts to **Nm**	*notch-transcription-initial-rate*	Chance that a single **N** will be produced
**Nm**	N associated with membrane, interacts with Dl from another cell, cleaved	**Nm** moves laterally from **Mem** to **Mem** **Nm** converts to **Nc** when across from **Dlm’** on another cell		
**Nc**	Cleaved N moves from cell membrane to nucleus	**Nc** converts to **Nn** when it reaches the nucleus	*notch-cleave-transport*	Sets randomness, direction and time frame of movement
**Nn**	Nuclear form of N, transcription factor	Ages out Alters **N** transcription Alters **Dl** transcription	*notchAge cleaved-nuc-notchcount*	Time period a **N** will exist Alters **N** and **Dl** initial transcription rates

Agents are represented in bold print. Model commands are in italics

**Fig. 2:**
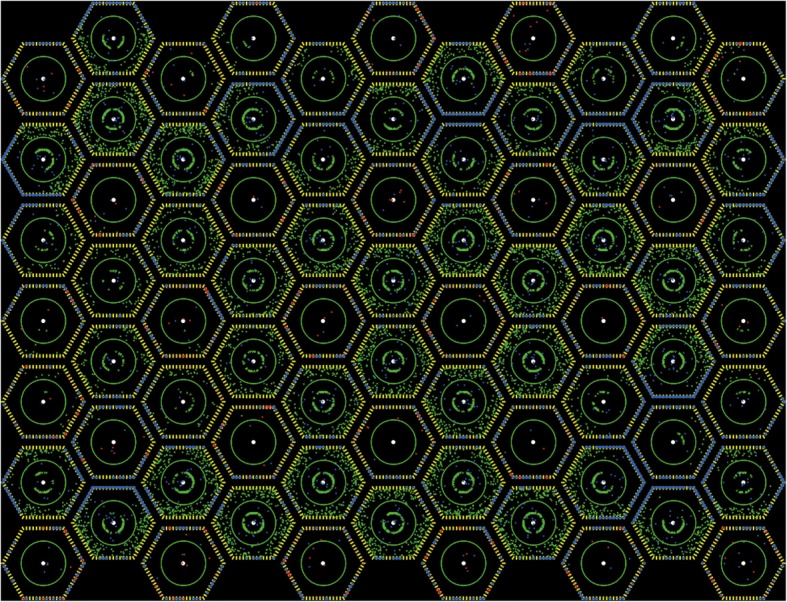
A NetLogo screen capture of the sheet of cells in the model space. The yellow agents are **Mem** and represent the membrane of the cell. **Nm** agents are blue, and all **Dl** agents are red. The nucleus is delineated by a green circle with a white dot in it (representing the spatial location where new agents are produced as part of the Netlogo programming environment). **Nc** agents are green and are outside the nucleus and the **Nn** agents are also green but are within the nucleus. They accumulate in an arc just inside the nuclear

Structural agents remain fixed. In our model, a hexagonal cell is represented by chain of structural agents, **Mem**, with each agent equivalent to a region of the lipid membrane. An agent called **Nuc** defines space in the center of the cell and the **Mem** and all other agents are assigned as belonging to a particular **Nuc** (Fig. 2). A two-dimensional array of these “cells” forms the model space, which approximates the cell geometry observed at this developmental stage [30]. Other models suggest that the cellular geometry may be crucial for appropriate patterning [31]. The array can be altered: structural variables developed for the model include cell and nucleus radius, lipid-density, and the overall shape of the sheet. The number of cells within a sheet can also be varied easily within the model. For the experiments in this paper, we ran the model with 77 cells. We chose this number of cells since it was computationally tractable, but also a model space where most of the cells were surrounded by six other cells (rather than three or four as seen near an edge). An average compute time for one run of the model was about an hour.

The second type of agents are active components representing various states of N and Dl that model the steps seen in Fig. 1. Agents **Dl** and **N** are generated at a variable initial rate and tagged as belonging to each cell’s **Nuc**. The **N/Dl** agents are assigned a random heading and move towards **Mem** agents and transition to **Nm** and **Dlm** as they associate with these agents (step 1). Both agents move randomly between **Mem** agents after production. **Dlm** is converted to an active form called **Dlm’** at a variable rate within the model (step 2). When **Nm** and **Dlm**’ agents are opposite each other on adjacent cells, **Nm** is converted to **Nc** (the first cleavage step, step 3). The average time of the second cleavage step and subsequent migration of **Nc** to the nucleus (the conversion of **Nc** to **Nn**) can also be varied within the model (step 4). The number of **Nn** agents provides feedback on the system, upregulating **N** and downregulating **Dl** average transcription settings.

Transitions between agents are mediated in various ways within the model. **N/Dl** is converted to **Nm/Dlm**, when they approach a **Mem** agent. Likewise, the **Nm** to **Nc** transition occurs when a **Nm** and **Dlm’** move to adjacent **Mem**s across from each other on the adjoining cell. In other cases, transitions are generated by rates that have stochastic features that model complex molecular interactions or transit in cell compartments. Such stochastic features are a property of biological systems and random elements of fate determination for the N pathway have been confirmed in vivo [25]. For example, the initial production of **N** and **Dl** are set as a variable that creates a distribution of values around a mean. **Nc** transitions to **Nn** using a random step feature with a rate set as an average distribution. The transition from **Dlm** to **Dlm’** can be manipulated by a setting that creates an average time but not an exact time for the transition. The stochastic features of the model are produced using pseudorandom number generator that is part of the Netlogo programming language (see methods). To prevent a simple buildup of agents over time, we also have an age-out feature for all N and Dl agents. Agent “death” is set relative to a birth date for an average number of ticks and then the agents are removed from the model space. The age-out feature models the biological lifespan of molecules. These transition variables can be set initially within the NetLogo model or specified by a driver program that directs multiple runs of the model.

### Running the model

We can observe a single run of the model using the NetLogo program; each agent has its own color designation and we can watch as the agents move within the model space (Fig. 2). Generally speaking though, we perform multiple runs of the same experiment of the model using a program that specifies a range of parameters and distributes the model runs across a cluster of computers. Production of agents, transitions, feedback and age-out occur in a specific order for each tick of a model run. The NetLogo program calculates two outputs from the model runs: an integer count of **Nn** agents (the signal level-**N** count) in each cell produced as a ordered string at each tick, and a raw count of the neurons at each tick. Neurons are defined as having a zero **Nn** count, while other cells are defined as nonneurons or skin cells.

### Analysis of model data

**Fig. 3:**
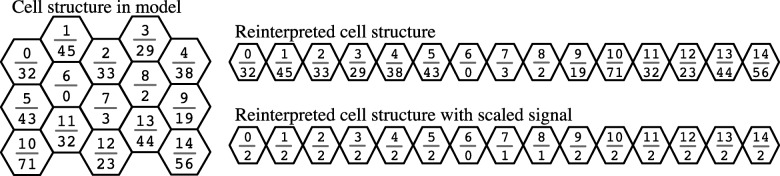
Remapping from the model representation to a sequence. As the model runs, two types of information at each time point are collected: The number of **Nn** agents in a given cell (signal strength, bottom number in each cell) and the position of that cell in the field (pattern, top number in each cell). The scaled signal condenses the N level to the base 10 logarithm with the special zero-signal case equating to zero (a 0 to 0, 1 thru 9 to 1, 10 thru 99 to 2, etc)

A data logging mechanism stores the signal levels for each cell at each tick to a text file as a number sequence. Staggered rows that represent the cells with a sheet are preserved in a two-dimensional hex matrix, creating a sequential string that can be simply remapped into the original structure (Fig. 3). The signal level is stored as either a raw or scaled value. The scaled value uses a base 10 logarithmic equation to map the N level to one digit with the special zero-signal case equating to zero (a 0 to 0, 1 thru 9 to 1, 10 thru 99 to 2, and so on), allowing us to map large scale rather than small scale change. Cells labeled as zero are designated as neurons for each tick of the experiment. This scaling has the advantage of reducing a large numeric value to a single character.

**Fig. 4:**
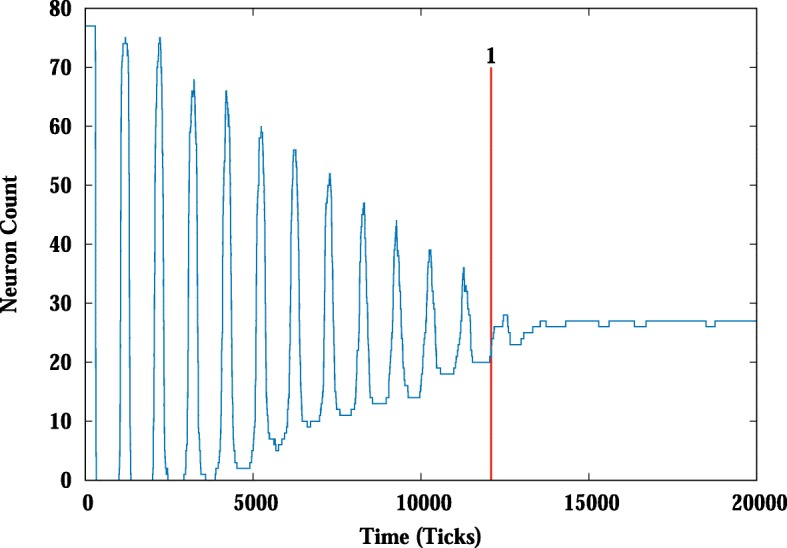
Stabilization time metric. Stabilization points are defined as the earliest point where there is deviation of one neuron or less as indicated by the red line

The dynamics of the cell population are analyzed first by simply looking at the signal levels. A graphing function plots the number of cells that have a zero signal-level (neuron) vs. ticks (time). Oscillation and then stabilization of the zero signal population is a consistent feature of many parameter settings within the model (Fig. 4). To capture this feature of the model data, we have developed a metric we call stabilization time. The stabilization time is calculated by looking at the deviation around the number of neurons at each time point over multiple time intervals. We calculate the standard deviation starting from the end of the data and moving towards the start. We define the stabilization time as the place where the variation in the number of neurons goes above one neuron deviation. In other words, the pattern is only varying by one neuron or less per tick (Fig. 4).

The developing pattern can also be analyzed dynamically since the number string can be remapped in a visual representation. It can also be reduced to a numerical value to make comparisons easier. A roset pattern, a central neuron surrounded by six epidermal cells in a repeating pattern, is the goal developmental pattern for the whole sheet of cells, with an optimal number of rosettes of 27 or 28 for 77 cells. Using the string that relates signal level to position, we defined rosettes as neurons with six non-neuron neighbors, and then accounted for edge effects by adding in edge neurons that also had an edge base optimum of non-neuron neighbors. Occasionally one imperfect rosette is generated at the edge, but rosette counts that stabilized and were within two neurons of the 27/28 optimum were consistently found to have the correct pattern over a sample of the data. Therefore, our measure of rosette counts is a good proxy for overall pattern.

Once rosettes and stabilization times have been determined, these measures are displayed on color-coded graphs with hot colors representing high numbers of rosettes and high stabilization times and cool colors representing low numbers for these measures. In addition, graphs that combine these measures were created to give an overview by assigning category designations and colors. In addition, dynamics can be measured by tracking change of signal levels. Using the string that related signal level to position, we can use a variation of Hamming distance (derived from information theory) to measure the amount of dis-similarity between two strings of codes at different time points. In traditional hamming distance, two binary codes are compared for differences and a count of these differences becomes the distance between the codes. With our approach, two strings are compared and a new string is be generated that flags any differences with an asterisk, representing where the two strings do not match and retaining the character code when there is no change. Thus, variation in time can be captured in a progressive way and used to characterize model stabilization during a run.

### Model output-single runs

**Fig. 5:**
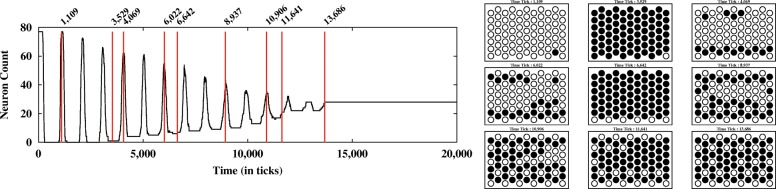
Dynamics of a single model run. A histogram is created to capture the neuron count vs time (left) as shown above in Fig. 4 and a diagram showing position can be constructed (right) from the **N** level and positional data information. White represents neurons and black, skin cells

**Fig. 6:**
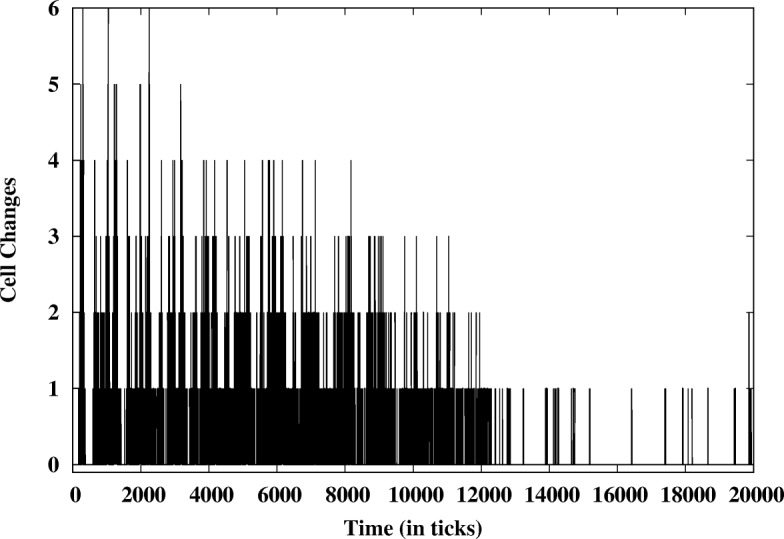
Measure of cell fate change over time during a model run. This analysis compares the changes in cell fate using a modification of Hamming distance to compare strings at adjacent time points

The model with the agents, transitions and features described above produces a stable rosette pattern with a variety of parameter settings (see below). In our observations of single model runs, the model produces an oscillating neuron count that then stabilizes, locking the system into the observed pattern (Fig. 5). During the oscillations, cell fate is changeable as the amount of **Nn** varies. Notch and its downstream targets have been shown to oscillate in vivo as well [32]. Cell interactions produce a pattern that is constructed and deconstructed, till a stable rosette pattern is produced. This is more clearly illustrated using a measure that tracks changes in cell fate (as determined by N level, neuron to skin or skin to neuron) over the time frame of the model (Fig. 6). As the run progresses, we see fewer changes in fate over time, suggesting the system stabilizes in steps or regions. Analysis of a subset of individual runs suggests that areas with stable patterns develop and then influence the rest of the sheet of cells towards the optimum rosette packing. So the fine grain interactions of the **N** and **Dl** acting between cells generate the larger scale patterns within the system.

### Conditions for model stability and patterning

Experiments were run to understand how the levels and timing of the components produce stable and correct patterning. There were basic four parameters that were varied in these initial experiments: initial transcription rates for both **N** and **Dl**, which controls the overall level of the components; and transition time for **Dlm** to **Dlm’**, and transition time for **Nc** to **Nn**, which controls the timing of component availability to the system. For each parameter set, 21–29 seeds were performed to assess the reproducibility of the results. These are the 4 parameters that most directly involve stochastic features of the model and that can be specified within initial settings. The initial transcription rates directly impact the level of the **N** and **Dl** family of agents, while the two transition parameters impact the timing of events in the model. Model features that are static and age-out were held constant for these sets of runs. The settings for this data set are summarized in Table 2.

**Table 2:** Model parameter settings

Parameter varied	Representation in the model	Settings examined
**N**	N agents initially transcribed	8 to 24 agents in increments of 2
**Dl**	Dl agents initially transcribed	8 to 24 agents in increments of 2
**Dlm** to **Dlm’**	Transition from form associated with membrane to form that interacts with N	0, 50, 100, 150 ticks
**Nc** to **Nn**	Transition from cleaved form to nuclear form	50, 75, 175, 225, 275 ticks

Agents are represented in bold print

**Fig. 7:**
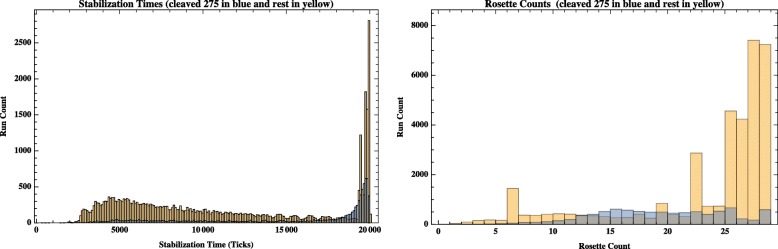
Overall characteristics of runs with variations of four parameter settings. The histogram on the left shows stabilization time for all runs, while the histogram on the right shows rosette counts for all runs

The composite characteristics of all runs are shown in Fig. 7 as histograms of all runs for stabilization time and rosette count. The model “works” for a large group of parameter settings (shown in yellow), where we define working as having a less than 18,000 tick stabilization time and a rosette count of greater than 25. In our initial experiments, one set of parameters does not produce stable or correctly patterned runs: the highest **Nc** to **Nn** transition setting of 275. These experiments produce runs that are inconsistent, with long stabilization times, no stabilization, or low rosette counts (shown in light blue). We do not present any further data on these settings in the results.

The other parameter settings do produce some or all runs where the model works. The shortest stabilization time is about 2500 ticks and this end of the data represents both runs that stabilize with the correct number and some that do not. There is a peak of runs that stabilize very late or not at all (about 30%). If a run doesn’t reach stability by 20,000 ticks, it is assigned that value. These runs are difficult to assess as a group since our rosette measure may inaccurately assess the pattern for runs that are still oscillating at the end of the 20,000 tick time frame. By looking at a sample of runs in this peak, we are convinced that some of these appear to converge on a stabilization time with good pattern beyond the 20,000 tick time frame of the experiment. In addition, there are some long time frame runs that appear to never stabilize and continue to oscillate with no suggestion of stability or that crash to produce either no neurons or no skin cells. The distribution of rosette then is skewed to the right, since many runs produce correct pattern.

**Fig. 8:**
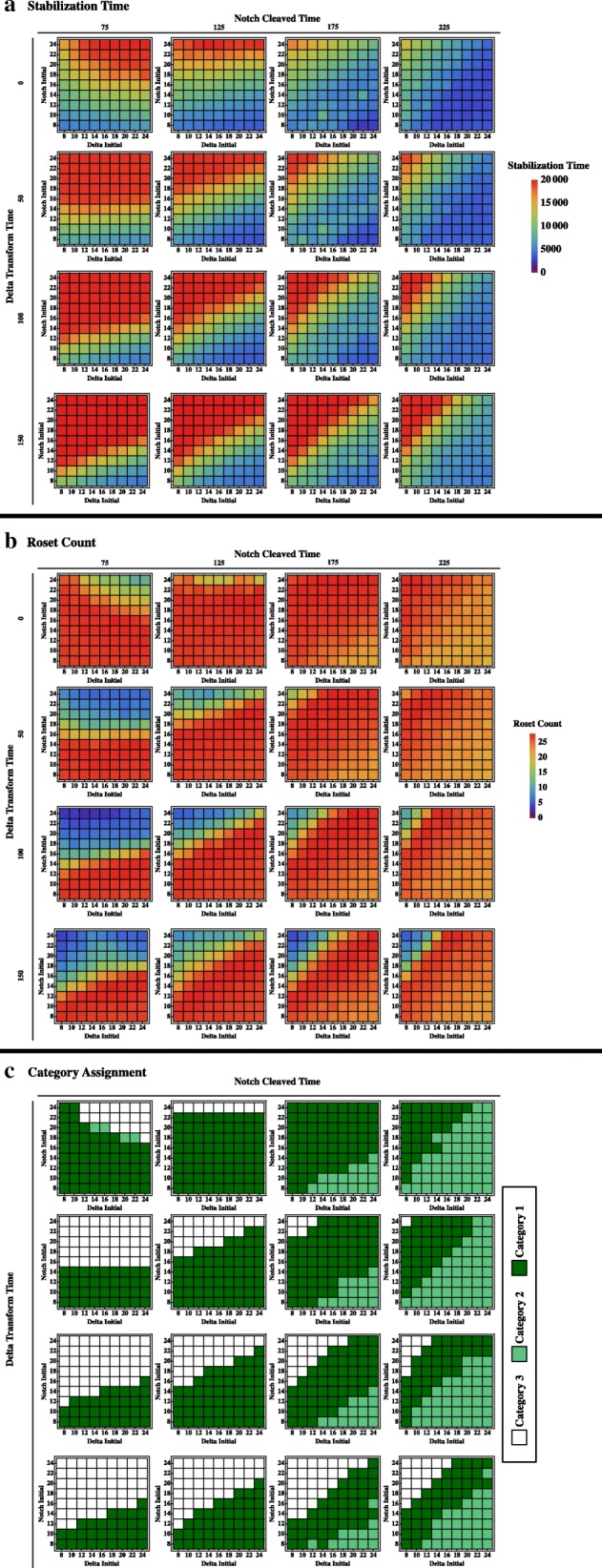
Data set varying four different parameters. For each individual graphs, initial **N** setting is on the y axis and initial **Dl** setting is on the x axis. Progression left to right for each row is increasing **Nc** to **Nn** transition time. Progression from top to bottom represents increasing **Dlm** to **Dlm’** transition time. **a** stability time (blue is low stabilization time and red is high) **b** rosette counts (blue is low rosette counts and red is high). **c** category assignments (white represents runs that fail to stabilize within 18,000 time, light green represents runs that stabilize with the incorrect number of rosettes (< 25), and dark green representing runs that stabilize with the correct number of rosettes

Figure 8 summarizes the data for experiments with 4 parameters. Stability time is shown at the top (Fig. 8a, blue is low stabilization time and red is high) and the middle set of graphs shows rosette counts (Fig. 8b, blue is low rosette counts and red is high). The bottom set of graphs shows the runs assigned into categories with white representing runs that fail to stabilize within 18,000 time, light green representing runs that stabilize with the incorrect number of rosettes (≤ 25), and dark green representing runs that stabilize with the correct number of rosettes. We define the dark green parameter settings as successful model runs-the model “works” for these parameter sets. We looked at the variability between runs/seeds for these successful parameter settings and found that in most cases, there is little variability. A successful run is reproducible, although the time it takes to stabilize may vary a little; the standard deviation for stabilization time for these experiments is less than 1000 ticks.

In comparing the three sets of graphs, there are several clear conclusions from the data. First, stability and pattern are not strictly correlated. High rosette pattern is found with a range of stabilization times. Some very short stabilization times do not produce good pattern. Systems that go through at least a few oscillations of **Nn** levels have a better probability of developing proper pattern. Second, stabilization time is strongly altered by the parameter settings, however many of the parameter sets generate rosette numbers representative of a proper pattern. It seems as if the model drives towards correct pattern and the parameter settings may largely determine when it gets to a stable place. Third, initial **N** levels are more important in determining a successful run than initial **Dl** levels, although both are important for stabilization time and pattern. Finally, the model has a sweet spot for the various parameters (best observed using Fig. 8c). For initial settings of **N** and **Dl**, the spot is on the diagonal, where there is close to equal levels of **N** and **Dl**. For the **Nc** to **Nn** transition, intermediate values again are best. **Dlm** to **Dlm’** transition is best with lower values. This tells us that there is an optimum timing for the system that involves all parameters.

The most dramatic effect on the model is obtained by varying the timing of the transition between **Nc** and **Nn**. This parameter would alter the timing of the availability of **Nn** to provide feedback within the cell and presumably alter the timing of oscillations in the system. Transition times of 125 and 175 ticks produce an overall sweet spot for the model. Decreasing transition time to 75 produces long stabilization times. Increasing transition time to 225 ticks only works when **Nn** initial levels are high. This reinforces our previous idea that longer transition times may work less well as they approach **Nn** age-out settings of 350.

The initial levels of both **N** and **Dl** are important since their agent numbers determine the probability of the **Nm** cleavage and subsequent effects on downstream transcription of the agent through feedback. The initial level is modified by the level of **Nn** at each tick. The best pattern and stabilization times are produced when initial **N** is low and initial **Dl** is greater than (for 75,125) or equal to (for 175, 225) initial **N**. It would seem that the level of initial **N** is most influential in setting up the stability properties for the system. However, the levels of initial **Dl** are not completely without consequence. Increasing amounts of initial **Dl** relative to initial **N** generates problems with pattern as seen in the higher **Nc** to **Nn** transition times.

The timing of **Dlm’** availability controlled by the **Dlm** to **Dlm’** transition time seems to somewhat impact stabilization time, with little impact on pattern. This was confirmed by looking at a small number of individual runs, most of which stabilize beyond 18,000 ticks with the proper pattern. The model works best when **Dlm’** is immediately available with longer availability times increasing model stability times.

An exception to these trends is present when both transition times are low (top left in Fig. 8c). High initial **N** requires less initial **Dl** to work. This is the only set of parameters where the majority of white box runs fail to stabilize based on a sampling of a small number of runs.

### Feedback within the model is essential

Feedback is an essential feature of the model. In the iteration of the model that produces successful stability and pattern, production of new agents is dependent of the level of **Nn** agents. High number increases **N** production and decrease **Dl** transcription and visa-versa. We used a subset of parameter settings to look at the impact of this feedback in the model. This is easy to do since the code that specifies these interactions can simply be removed. The only version of the model that works properly involves feedback of **Nn** agent levels to the producer that controls both **N** and **Dl** transcription. The results are presented in Table 3 below. When either feedback is removed, no or low numbers of neurons are present in the cell sheet. It may be that **N** or **Dl** regulation may not be important in a given cell, but is necessary within the system to produce the neuron fate.

**Table 3:** Results of Feedback experiments

Experiment	Outcome
**Nn** turns up **N** transcription and turns down **Dl** transcription	Good stability and pattern across a wide set of parameters
**Nn** turns up **N** transcription only	Model crashes with 0 neuron count and fails to oscillate
**Nn** turns down **Dl** transcription only	Low neuron and rosette count (< 5), no oscillating runs that stabilize
No feedback	Model crashes with 0 neuron count and fails to oscillate

Agents are represented in bold print

## Discussion

We have created an agent-based model (ABM) that simulates the molecular components for the **N** signaling pathway as agents acting within a spatial representation of a cell. Using a set of assessment tools, we show that the current model accurately reproduces the rosette pattern of neurons and skin cells in the system over a wide set of parameters and looked at the impact of levels, timing and feedback for the N and Dl components of the model. The data presented in this paper looks at aggregated aspects of the system, like number of neurons and pattern.

**Fig. 9:**
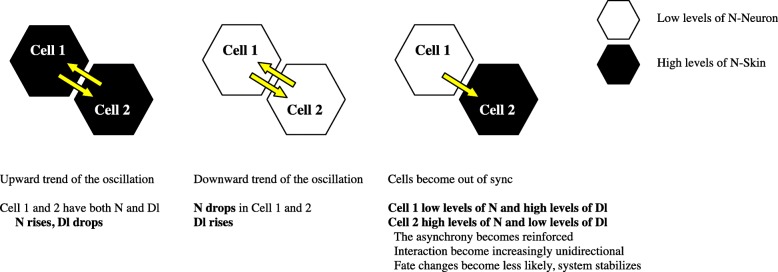
Interactions within model that lead to stabilized fate

We can postulate from our data the likely events that lead to larger fate decisions (Fig. 9). At the first level of complexity, initial values of **N** and **Dl** produce a certain number of **Nn** that turn up **N** and down **Dl** in each cell across the grid. As **Nn** numbers climb, **Dl** components decrease in each cell across the grid. Without **Dlm’** in adjacent cells, **Nm** is not converted to **Nc**, and **Nn** numbers decline and it ages out, which leads to decreased production of **N** and increased production of **Dl**. Once the levels of **N** and **Dl** even up, the process starts again. We expect that as the process goes on, adjacent cells become out of sync in terms of their oscillations. The stochastic nature of the system would ensure that the behavior in each cell is not identical, leading to this asynchrony. A cell (call it *cell1*) **neighboring** a cell with high **N** (*cell 2*) is more likely to become a neuron, since the high **N** cell would have low **Dl**. *Cell 1* then would soon develop low levels of **N** and high levels of **Dl**. After a few rounds of oscillation, these patterns become reinforced and fate change becomes less likely (As observed in Fig. 6). As *cell 1* accumulates more neighbors that have high **N** values (cells 3–6) and low **Dl** values, both cell types become locked in. The arrangement of one in the middle of six would appear to optimize the self-patterning of the system. Based on this model of events, the “lateral inhibition” that is often cited as a hallmark of this model is really the absence of **Dl** on adjacent cells rather than any inhibitory property of **Dl**. These hypotheses can be directly tested in our existing model by looking at the oscillation patterns of individual cells under different conditions and that work is in progress.

We expected that the initial rates of both **N** and **Dl** agents would be an important feature of the model since the level of these protein products in the biological system would determine the probability of the N cleavage and subsequent effects on downstream transcription. Based on the hypothesis presented above, different ratios of **N** and **Dl** agents would change the timing of the oscillations, as well as having some impact on pattern. Indeed, increased **N** levels result in longer stabilization times and larger oscillations. For a given level of **N**, **Dl** level does not have a big impact on stability or pattern, except when there is a considerable mismatch in levels.

The **Nc** to **Nn** transition was most influential in the outcomes from the model, suggesting timing is important to the formation of system pattern. Biologically, the transition **Nc** to **Nn** consists of two cleavages, one at the cell membrane and a second an endocytic compartment that releases a fragment that can then enter the nucleus and alter transcription [21, 22]. We simply represented those multiple steps as one transition, and represented this transition as a timing variable. This parameter interacts with other features of the model in interesting ways. Long transition times interfere with the action of **N** and essentially limit feedback in the model. Short transition times cause faster oscillations, but also long stability times, most likely because the chance for substantial asynchrony to develop between cells would be limited and the system swings back and forth with little progress in fate assignment. Within the sweet spot for this transition, we see more of the effects of other parameters.

Feedback is absolutely necessary for the model to work. This is expected since it is the chief mechanism by which change in the system occurs, but our model suggests that feedback is required for both **N** and **Dl** components. Although, there is limited evidence that N regulates its own transcription or Dl transcription, such feedback has been seen in a few instances and has been postulated to part of this regulatory circuit [1, 10–13]. Feedback to N or Dl transcription may be directly performed by N or by one of the downstream genes, but our model confirms its importance for the biological decision-making carried out by the system.

### Model validation

Many of the experiments that have been done over the last 20 years have been aimed at discovering the biological players in the system and the downstream components necessary for the expression of cell fate. The focus has been on the cell as the unit of function, and molecular biology experiments have created complete loss of function or gain of function for the system. In most cases, the pattern has been completely disrupted, generating all neurons or all skin cells. Less emphasis has been placed on altered pattern from these early developmental steps.

However, several outcomes of this model are supported by the existing literature. The model mirrors the findings of much of the literature that manipulation of N levels and its processing alone can impact both fate and pattern. The stoichiometric nature of the N pathway had been postulated from genetic experiments since the N locus is both haplo-insufficient and triplo-mutant (both ½ dose and 3 doses lead to a altered phenotype) and our model findings are consistent with that interpretation [1]. Genetic data suggests that a ratio of the levels of the N ligand and its Dl receptor is important in fate and in establishing an asymmetry in the levels of these proteins that develop over time through feedback loops [1]. Our model is consistent with the idea that both initial and relative levels of these proteins over time determine both fate and pattern and that the process is dependent on feedback. Our model also produces oscillations of these components. These oscillations have been observed in the biological system for N protein levels and downstream targets [32, 33].

We believe there are further experiments that could be done to validate the model. Levels of N and Dl protein might be controlled through RNAi constructs in a mutant background or using antibodies or small molecules that modulate signaling [33–36]. There are chemical inhibitors of the proteases involved in the cleavage step that could be used at varying dosages to produce alterations in the efficiency of the process [37, 38]. The pattern can be monitored using antibodies targeted at various nervous system molecules and a technique has been developed to look at oscillations of the downstream proteins Hes 1 and 7 [33]. These types of experiments are necessary for further validation of the model. The N signaling system produces other patterns beside rosettes and one of the challenges will be to see if our model can also produce those other patterns and how we might produce them with the basic components we have already incorporated.

### How our model compares to other models

Several models of the Notch signaling pathway have been created previously. Waddington was one of the first to look conceptually at the process of fate determination as that of the cell moving on an energy-landscape molded by the effects of the transcribing genome [15, 16]. Waddington represented these influences as a system of differential equations implementing the continuous effects of the various interacting genes. Many of the current models have followed suit, by implementing individual cells in the population as compartments within a differential equation system. The Collier model is the most successful of these recent efforts with the general idea of the model modified and improved by others [17–19, 24, 39, 40]. Initially analytical and numerical analyses focused on system dynamics while some later work extended the Collier results, focusing on the molecular interactions between cell compartments [18]. The Marnellos group relied exclusively on numerical analysis, but used an evolutionary optimization technique to tune the model dynamics [19]. Finally, a modeling approach has been used to simulate genetic expression state propagation across cell populations in the spinal nervous system during vertebrate development [41]. All of these models show proper pattern of the basic rosette structure. A recent modification has allowed the model to be extended to produce other patterns associated with N signaling [24]. Other approaches to N pathway modeling include gene-regulatory networks [20, 21], extended Boolean Networks [22], and discrete stochastic processes [23]. One recent model that uses a differential equation approach takes into account the volume and geometry of the cells making up the system and shows the impact of these factors in producing pattern [31].

While all of these models represent the patterning of the N signaling system well, they focus on either the interactions between cells and those pattern dynamics, or they look at the development of fate choice within the cell. None of them integrate the three levels of hierarchy inherent in our model. These models are also harder to manipulate than ours, often requiring altered or additional equations to ask experimental questions. Some of the models assume a role for inhibition in the model, while our model allows the molecular interactions to illuminate the process. Our modeling environment is robust and does not require a precise description as seen in the differential equation based systems. Overall, our model represents the biology better, where the overall behavior of molecules is not precise and systems can often handle some deviation.

Several authors have suggested that ABM may be a fruitful approach to modeling biological systems. Walker and Southgate (2009) suggest that spatially hierarchical models such as agent-based systems are a good approach to modeling biological phenomena at multiple scales [28]. However, they state that few multi-scale models had been developed as of that time. Bartocci and Lió (2016) suggest that agent-based models are well suited to understanding how cellular interactions produce systems characteristics [42]. Richmond et al. (2010) in an earlier paper had made similar points about the utility of agent-based models and developed a modeling environment using an agent-based program call FLAME that runs on both single and clustered systems and provides a template for these types of modeling experiments [43]. Our choice of ABM and the NetLogo programming environment embrace the advantages laid out by the authors in these papers and facilitates a multi-scale analysis of this developmental process. It allows us to easily manipulate components and the processing steps associated with the pathway, and make it easy to add more steps into the model. For example, we have already built the model with **Nm** and **Dlm’** cis inhibition interactions that are observed in the biological system [44]. **Dl** in this case acts to sequester **N** from intramembrane cleavage, essentially making too much **Dl** a detriment to the system. We look forward to confirming additional biological features in this system.

Our model is based on an understanding of the components and their actions discovered through traditional methods. It is strongly based in the biological literature, although we have made choices and assumptions on implementation. The hypothesis-driven framework creates an internalized reconstruction of the subcellular process and an external analysis of the system dynamics that allows for an integrated exploration of the role of the subcellular in the multicellular pattern. In addition, a fuller understanding and a formal description of how our model generates this dynamic pattern may inform computation problems. The N signaling system and its biological approach to pattern formation inspired an algorithm that addresses a key problem in distributed programming [25]. The flow of information between individual components in a distributed system as presented by our model may be used in a similar way to approach these problems in computational systems.

## Conclusions

We have created an agent-based model that simulates the molecular components of the N signaling pathway within representational cells capable of creating a multicellular pattern consistent with what is observed in the biological system. The model has 3 levels of complexity: the specific timing and level of each molecular component within each cell, the interactions between cells, and the formation of pattern across the system. The signaling pathways within and between cells in our model interact in real time to create a spatially correct field of neurons and skin cells. The model produces a stable correct pattern for the system under a variety of model parameter settings. We found that the dynamic timing and availability of **N** and **Dl** components of the system were central to the formation of a cell fate and a correct and stable pattern. Levels of the **Nn** agent oscillate up and down within individual cells and in the system. Positive feedback to **N** levels and negative feedback on **Dl** levels provided by **N** levels over time are essential to the model. According to our model, cells that have high levels of **N** and low levels of **Dl** engage with neighbors that have low or no **N** and high **Dl** levels, stabilizing these cells into their fate. The timing of oscillations between neighboring cells most likely establish stable fate at the middle level of complexity and the construction and deconstruction of pattern is necessary for the whole field to stabilize correctly, since model runs that have short stabilization times often do not have correct pattern. Therefore, the components **N** and **Dl** control cell fate, neighboring cell fate, and the larger pattern. This model can be used to make predictions about the N signaling system, but can also be used to elucidate general rules of biological self-patterning and decision-making.

## Methods

### Model elements

NetLogo is a modeling tool developed following in the long tradition of Logo and directly descended from StarLogo [16, 24]. Conceptually, NetLogo allows for the visualization of agents that are autonomous software entities embodied in a virtual environment that are capable of navigating, sensing, and manipulating that environment, including other agents. These agents are called turtles, following from Logo, but can be assigned to a specific population called a *breed*. Agents are akin to objects in an Object Oriented (OO) language. These populations can be called upon to perform different actions and agents can create other agents, allowing for a mechanism fundamental to the model where one agent produces a range of agents to construct a sophisticated architecture. *Globals* set up the initial starting features of the environment and the placement of the agents within that environment as well as configure model output. The program is constructed in a modular fashion as described in the following sections. The complete code for the model and driver and the data for the experiments in the paper can be found in supporting files as follows: Additional files 1, 2, 3, 4, 5 and 6.

#### Globals

There are four categories of globals: *configuration*, which sets up the initial structural features of the model environment such as cell radius and unit move based on that radius; *model,* which governs the actions of the agents within the model; *general*, which sets up how information is collected during model runs; and *reporting*, which directs the model output.

#### Agents

The model is constructed from four general types of agents (**Nuc**, **Mem**, **Dl** and **N**) with a total of nine different breeds within the programming environment (Table 1). **Nuc** and **Mem** are structural components that provide the major organizing features for the model. The **Nuc** breed provides points of orientation for cells in the structure and act as factories for production of **Mem**, **Dl**, and **N** agents. These agents are then linked with their progenitor **Nuc** (their parent). The **Mem** breed is more complex. The **Mem** represent the lipid membrane that form the walls of the cells around a parent nucleus, but also form the basis of locations within cells and in interactions between cells. The placement of the **Mem** is determined by a cell radius setting from the configuration global, and the number and spacing of the **Mem** agents can be altered to impact the granularity of the model. The **Nuc** and **Mem** agents are put into place during setup procedures and do not change during the model run. **N** and **Dl** agents are breeds that are dynamically created and destroyed during model run, providing the signaling mechanisms in the model (Table 1). There are 3 different Dl category breeds (**Dl**, **Dlm**, **Dlm’**) and 4 different N category breeds (**N**, **Nm**, **Nc**, **Nn**) that represent the transitions of these proteins during signaling (Table 1). **N** and **Dl** agents move through the model environment with their location in the model tracked and their spatial association with other agents often results in one breed being transformed into another.

### Procedures within the model

#### Setup

The model is initiated through a *setup* procedure that clears the model space, and initiates globals and model settings that are variable. In general, the model takes direction from a head program (described below) that defines specific parameters and the range of parameters that will be tested within an “experiment” and then initiates the *Go* command below. Each experiment is given a unique designation or seed. Setup then initiates the **Nuc** breed and lays out a sheet of cells with **Nuc** and their children **Mem** agents in a hexagonal pattern. Relative position of each cell is determined in the sheet via the *build-neighbors* command that groups a set of detailed commands responsible for assigning agents to a specific parent **Nuc** and then determining neighboring agents and their parents. Action within the model takes place within the context of this sheet of cells. Different layout commands implement cell topology within the sheet, nuclear topology and cell radius.

#### Go

Once the model is setup, a Go command initiates each model run. The Go procedure tells the model to perform several steps:

1. Remove components that have hit an age limit.
2. Ask each nucleus to produce some number of **Dl** and **N** agents.
3. Move existing **Dl** and **N** breeds within the system, depending on their type.
4. Allow agents to manipulate neighboring agents if applicable.
5. Increment time. Time begins for the model at *t* = 0.

Step 1: The age-limit (age-out) for **N** and **Dl** agents are set as one of the initial model parameters. This age limit removes agents from the system and is dependent on breed type. Thus, when a **Dl** agent is created the clock begins and is never reset even though it goes through a set of breed transformations. Regular destruction of agents is essential for the model to run, since the definitions within the model for a neuron is based on a zero **N** count, which would never be achieved if components did not “age-out.”

Step 2: At each tick, additional **N** and **Dl** agents are added to the system. The initial agents are **Dl** or **N** breeds. There is an initial transcription setting for these components specified by the head program and then each additional tick brings a reassessment of the transcription rate based on the amount of **Nn**. The initial setting establishes a mean for production of agents, and variation around that mean is based the number of **Nn** and on a pseudorandom number generator that sets the value of that rate (described below).

Step 3: Agents move and transition between breeds.

- The **N** and **Dl** agents move in a stepwise fashion to the membrane, moving one patch space per tick with the distance defined by the cell and nucleus radius.
- When **N** and **Dl** agents move within one patch distance occupied by a **Mem** agent, they transition to **Nm** or **Dlm** respectively.
- **Dlm** agents transitions to the **Dlm’** breed based on a parameter setting for the transition
- **Nm**, **Dlm** or **Dlm’** agents move laterally from **Mem** agent to **Mem** agent.
- Once **Nm** is converted to **Nc** (see step 4), **Nc** then moves from the membrane to the nucleus. These movements are encoded as a *diffuse-proteins* command, where each agent has code related to their movement (See below).
- **Nc** agent within 1 patch of the nucleus transition to **Nn**.

Step 4: This is the most complex part of the model. In the current iteration of the model, this step is about the transition of **Nm** to **Nc**. In our implementation, the transition is coded to look at positions of **Dl** associated with **Mem** agents on a neighboring cell in relation to the position of **N** associated with **Mem** agents on the home cell. If a **Dlm’** is within a defined set of locations across from a **Nm** agent, then **Nm** transitions to **Nc**.

Step 5: This step moves forward the model timing mechanism and assigns a tick number to all events within the model.

### Stochastic features of the model

The model is designed to implement random features to ensure that a regular ordering of events does not introduce artifacts into the results and to match the stochastic nature of the biological system. The stochastic features rely on a pseudorandom number generator using the Mersene-Twister algorithm implemented in NetLogo [45]. Random agent selection is implemented when a specific set of agents is required to act, and randomized action selection is implemented when there is a range of possible choices. Random agent selection is used primarily to ensure that order of agent selection does not bias the results in the favor of one agent or another based on one agent always performing its action first. Random action selection is based on the notion that proteins have the Markov property in that they do not retain a memory of previous actions upon which to make future decisions beyond their current state. Through a combination of these two random features, the model should prevent errors from being introduced due to one component having greater privileged over other competing components.

Much of the biological “noise” of the model is implemented using the *random* command feature of NetLogo. Each run of the model is seeded, which supplies a series of pseudorandom values that are used to generate variable parameters of the model. Parameters varied in this paper have some “noise” incorporated. Initial transcription and subsequent transcription rates of **N** and **Dl** are randomly generated around a mean setting. Transition between **Dlm** and **Dlm’** has similar stochastic components. The movement of **Nc** towards the **Nuc** has a random component since **Nc** agents use the *diffuse-protein* command for movement. This command directs movement towards the nucleus based on a Gaussian distribution around the parameter setting for the **Nc** to **Nn** transition.

### Driver program

A driver program provides an isolated execution environment between individual runs, stores model output as a set of nested directories based on the parameter space, and captures results of how the model was run, to allow for later examination and confirmation of the execution environment. The driver program functions by running and manipulating the model in headless-mode (without visualization) within the Java Virtual Machine (JVM), which is a designed feature of NetLogo. Once the JVM is started, Java classes are loaded into the environment, allowing the driver to load the model as if a human user were working with the software directly. Once running, the driver iterates, in a nested order, the five parameters we varied in this paper: Notch transcription initial rate, Delta transcription initial rate, Delta membrane transform time, cleaved Notch diffusion time, and random seed. This implementation starts with a directory named for the first value of notch transcription initial rate, which will contain all other combinations of the remaining parameters. At the bottom of the directory structure is a directory identifying the random seed. The nested directory structure can be later merged with similarly structured directories. This allows for a larger data-set to be built incrementally from small sets of data. This also allows for a common structure to analyze and aggregate data for later assessment of model behavior and performance.

### Output from the model and measures

#### Data logging

From each model run, two files are generated: notchNucCounts.txt, and neuronCnts.txt. The file notchNucCounts.txt contains the individual cell signal levels. The file neuronCnts.txt contains the count of neurons (based on a zero signal-level metric) of the system for each time tick as two columns. These basic files are then manipulated to produce the seed or aggregated data. This data then is pulled into graphing programs to create the graphs represented in this manuscript. See Additional file 6 for more information.

#### Stabilization time

This measure is determined using neuronCnts.txt and scanning from the last time point to identify the place where the neuron count for a given run does not vary by more than one neuron. The earliest tick frame where that occurs is the stabilization time.

#### Difference assessments

Difference assessment provides a different perspective on stabilization of the model. Each tick produces a string of data that catalogues the number of **Nn** agents in the each cell, notchNucCounts.txt. The strings are compared using a Hamming distance function. The number of changes over time is then graphed. Changes over time can be mapped for an individual run or an aggregate set of runs.

#### Pattern

Pattern is assessed using a rosette count. For a rosette to be tallied, a neuron must have 6 neighbors that are skin cells, except in the case of edge neurons, which have an expected number of neighbors based on their position. For every cell, the numbers of the surrounding cells are catalogued and then the comparisons are made on a tick by tick basis or at the time end point of 20,000 ticks.

#### Category assignments

Category assignments are written as a set of if statements:

- Step 1. Is the average stability time less than or equal to 18,000, goes to Step 2
- *If not goes to cat 3, white boxes*
- Step 2. Is the rosette count greater than 25, goes to Step 3
- *If not goes to cat 2, light green boxes*
- Step 3 All remaining run are assigned cat 1, dark green boxes

#### Graphing

Graphs are produced using Mathematica or Gnuplot. Individual graphs for experiments are assembled into a grid.

## Additional files

Additional file 1: Netlogo Model. (TXT 28 kb)

Additional file 2: Driver Program. (TXT 10 kb)

Additional file 3: Stabilization Data (aggregated) for all parameter sets. (CSV 731 kb)

Additional file 4: Rosette Counts (aggregated) for all parameter sets. (CSV 641 kb)

Additional file 5: Hamming Statistics for all parameter sets. (CSV 145 kb)

Additional file 6: Data Paths for Model Analysis. (PDF 111 kb)
