# Supplementary figures and images for: An agent-based model of the Notch signaling pathway elucidates three levels of complexity in the determination of developmental patterning
Source: BMC Syst Biol. 2019 Jan 14;13:7. doi: 10.1186/s12918-018-0672-9 (PMC6332573; doi:10.1186/s12918-018-0672-9)

Stabilization Calculations

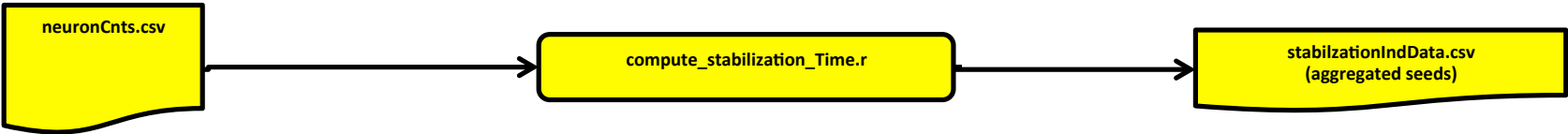

Structure Parsing

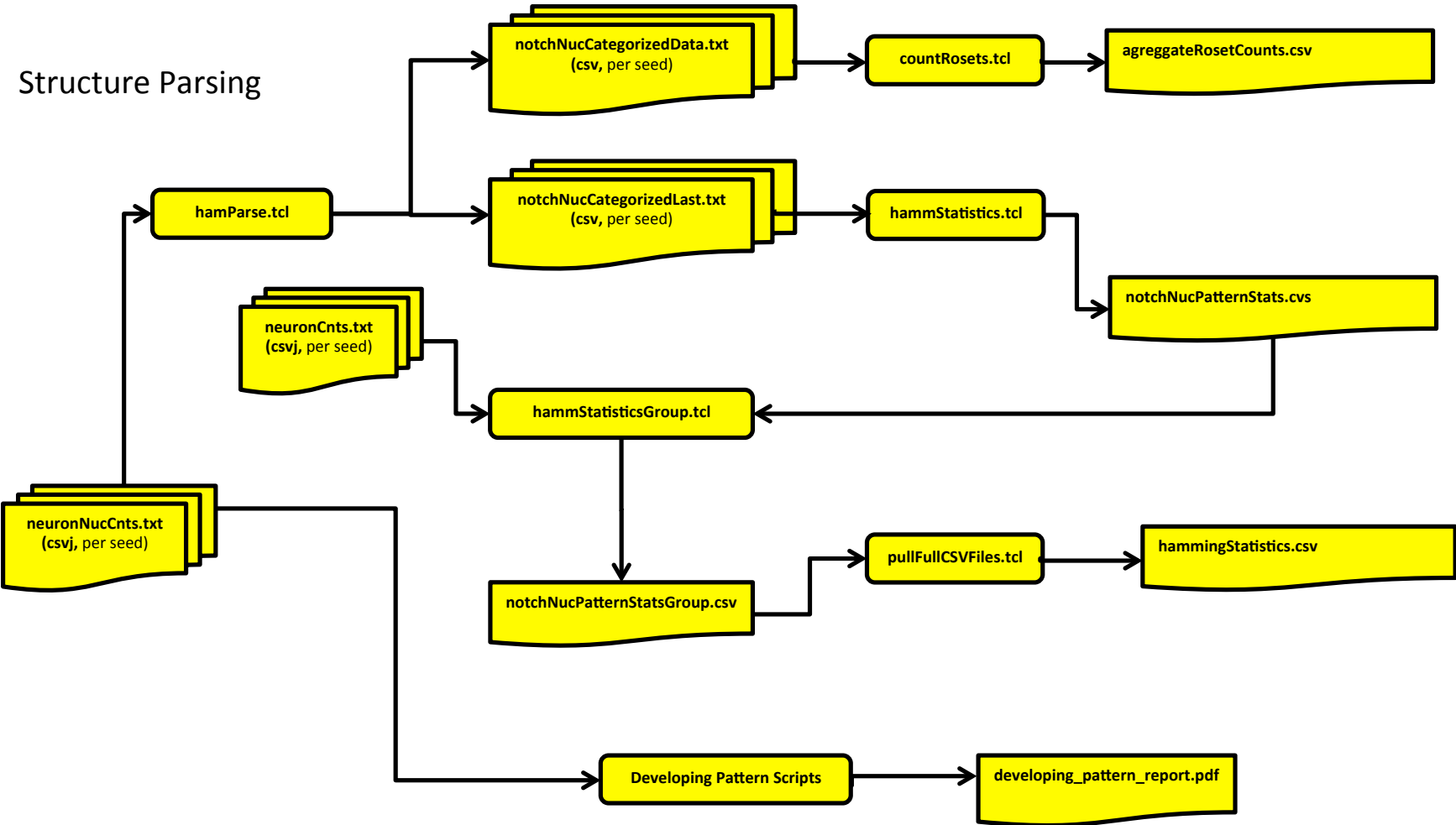

Aggregate Graphs

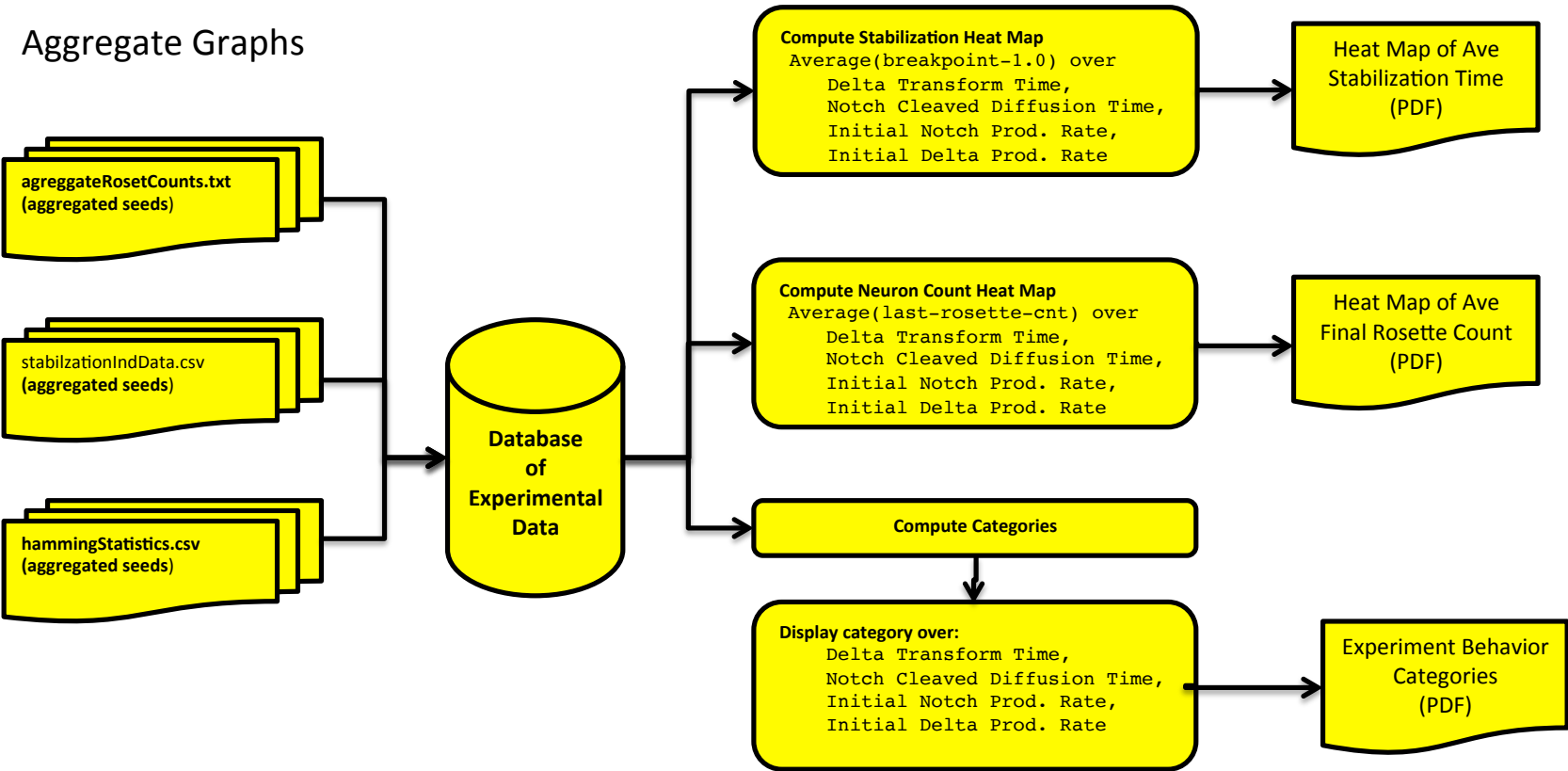

Supplement: Supplementary file 6 — Data Paths for Model Analysis. (PDF 111 kb) [file 12918_2018_672_MOESM6_ESM.pdf]
